# Long-term straw return with moderate nitrogen levels reshapes soil *bacterial* communities in a vertisol

**DOI:** 10.3389/fmicb.2025.1554657

**Published:** 2025-02-24

**Authors:** Zichun Guo, Rui Qian, Wei Li, Tianyu Ding, Lei Gao, Xinhua Peng

**Affiliations:** ^1^State Key Laboratory of Soil and Sustainable Agriculture, Institute of Soil Science, Chinese Academy of Sciences, Nanjing, China; ^2^Key Laboratory of Mollisols Agroecology, Northeast Institute of Geography and Agroecology, Chinese Academy of Sciences, Harbin, China; ^3^University of Chinese Academy of Sciences, Beijing, China; ^4^Crop Research Institute, Anhui Academy of Agricultural Science, Hefei, China; ^5^University of the Chinese Academy of Sciences, Nanjing, China; ^6^State Key Laboratory of Efficient Utilization of Arable Land in China, Institute of Agricultural Resources and Regional Planning, Chinese Academy of Agricultural Sciences, Beijing, China

**Keywords:** nitrogen fertilization, straw incorporation, soil microbial diversity, crop productivity, sustainable soil management

## Abstract

**Introduction:**

Incorporating straw into the soil is a sustainable practice that can mitigate some of the adverse effects of excessive N fertilization on soil structure degradation and microbial diversity reduction.

**Methods:**

This objective of this study was to determine the combined effects of straw management (straw return and straw removal) and N fertilization (0, 360, 450, 540, 630, and 720 kg N ha^−1^ yr.^−1^) on crop yields, soil properties, and soil microbial communities in a long-term wheat-maize cropping system.

**Results and discussion:**

The results showed that moderate N application (N450–N540) with straw return optimized wheat (283.5 kg ha^−1^) and maize (346.5 kg ha^−1^) yields, whereas higher N fertilization (N630, N720) led to soil acidification (pH decline of 0.51–1.67 units), irrespective of straw management. Straw return increased soil organic carbon (SOC), total nitrogen (TN), nitrate (NO_3_^−^-N), and available potassium (AK), but decreased ammonium (NH_4_^+^-N). Bacterial diversity increased at moderate N rates but decreased at higher N rates. Fungal diversity was generally higher under straw removal, with Chaetomiaceae increasing under straw return, whereas Mortierellaceae and Trichocomaceae declined at high N levels. The Mantel test showed a strong correlation between soil pH and bacterial diversity, while fungal composition was influenced by SOC, TN, and NO_3_^−^-N. Partial Least Squares Path Modeling (PLS-PM) demonstrated that N fertilization directly and indirectly increased wheat yield through improved soil properties, while straw return enhanced bacterial diversity, indirectly supported wheat yield. This study highlights the importance of balanced N fertilization and straw incorporation in maintaining bacterial community structure, fertility, and long-term crop productivity in intensive cropping systems on Vertisol.

## 1 Introduction

Nitrogen (N), an essential nutrient crucial for plant growth and development (Govindasamy et al., 2023; Huang et al., 2023), significantly influences cereal crop yields and straw production, particularly in wheat and maize systems. However, excessive N fertilization can degrade soil structure (Haynes and Naidu, 1998; Guo et al., 2022), and reduce microbial diversity (Chen et al., 2023; Qiu et al., 2023; Li et al., 2024), thereby threatening the sustainability of agricultural practices. Conversely, crop straw, a resource rich in carbon (C), nitrogen (N), and organic matter (OM) (Li et al., 2018), has been shown to enhance soil fertility and structure (Zhao et al., 2020; Liu N. et al., 2021), mitigating some negative effects of excessive N input. Incorporating straw into the soil is thus regarded as a sustainable management practice that can enhance soil microbial diversity (Zhang et al., 2021; Xu et al., 2023; Zhang et al., 2024). Nevertheless, in winter wheat-summer maize cropping systems, the slow decomposition of maize straw can limit its benefits and may even necessitate higher N inputs compared to straw removal to maintain wheat yield (Kong, 2014; Islam et al., 2022).

Recent studies have investigated the effects of straw management and N fertilization on soil properties, microbial communities, and crop yields, and found significant differences between different soil types and cropping systems. For instance, Li et al. (2019) demonstrated that a two-year straw return combined with a modest N input (270 kg N ha^−1^ year^−1^) enhanced soil organic carbon (SOC) and microbial activity in Fluvisols under a winter wheat-summer maize system. In contrast, Yang et al. (2022) observed that while similar practices improved soil fertility in Plinthosols under a spring maize-autumn maize system, they had limited effects on bacterial and fungal community structures. More recently, Liu et al. (2024) found that applying 225 kg N ha^−1^ yr.^−1^ with straw during the rice season in Inceptisols under a winter wheat-summer rice rotation significantly increased SOC, soil structural stability, microbial community health, and rice yields. However, these studies focus on a limited range of conditions (only with or without N addition treatments) or a narrow range of N application rates (from 0 to 360 kg N ha^−1^ yr.^−1^). As a result, the impacts of straw management and high N application rates on soil microbial communities in the wheat-maize cropping systems remain poorly understood.

Vertisol, known locally as Shajiang black soil, covers about 4 × 10^6^ ha in the Huang-Huai-Hai Plain of China. This soil type is characterized by low SOC and a poor structure, which limit soil tilth and crop productivity (Guo et al., 2019; Wang et al., 2021). Despite these challenges, Vertisol is one of the most important soils for grain production in China, supporting intensive wheat-maize cropping systems. To achieve high yields, farmers in this region usually apply N fertilizers at a rate of 300–800 kg ha^−1^ yr.^−1^ (Guo et al., 2022). However, there is an urgent need to clarify how N application rates and straw management practices influence the soil microbial community in Vertisol. This study investigates the combined effects of straw management and varying N application rates on crop yields, soil properties, and the composition and structure of soil microbial communities in a wheat-maize cropping system. The specific objectives are to: (1) evaluate the effects of straw management and N application rates on wheat and maize yields, (2) assess the changes in soil properties under the different treatments, (3) analyze the diversity and composition of soil bacterial and fungal communities in response to straw management and N fertilization, and (4) explore the relationships among microbial communities, soil properties, and crop yields.

## 2 Materials and methods

### 2.1 Study site and experimental design

A long-term field experiment was established in 2008 at the Agricultural Science and Technology Demonstration Center in Mengcheng County, Anhui Province, China (33°09′N, 116°33′E). The site has a typical monsoon climate, with a mean annual temperature of 16.5°C and annual precipitation of 900 mm. The soil, known locally as Shajiang black soil (Vertisol, USDA Soil Taxonomy), has a clay loam texture (31.5% sand, 38.0% silt, and 30.5% clay), and is derived from fluvio-lacustrine sediments. The initial SOC of the topsoil (0–20 cm) contained 8.22 g kg^−1^. The cropping system included winter wheat (*Triticum aestivum* L.) and summer maize (*Zea mays* L.).

The experiment included two straw management practices (straw return and straw removal) and six N fertilization rates (0, 360, 450, 540, 630, and 720 kg N ha^−1^ yr.^−1^). Each treatment was replicated three times, with each plot measuring 21.6 m^2^ (5.4 m × 4 m). The detailed amounts of fertilizers applied are shown in Table 1. Approximately 55% of the N for wheat and 45% for maize was applied as a basal fertilizer before tillage, while the remaining 45% (wheat) and 55% (maize), respectively, were used as a topdressing during the growing season. The maize was planted in mid-June and harvested in late September, while the wheat was sown in early October and harvested in late May. The cultivars used were Jimai 22 for wheat and Zhengdan 958 for maize. The seeding rates were 225 kg ha^−1^ for wheat and 30 kg ha^−1^ for maize. Crop yields at harvest were measured based on a moisture content of 14% across the plot (Guo et al., 2022). After each annual harvest, the straw was chopped into pieces of ≤10 cm which were incorporated into the soil to a depth of about 15 cm in the straw return treatments, while all straw was removed from the field in the straw removal treatments.

**Table 1:** Experimental treatments and application rates of inorganic fertilizers (kg ha^−1^) for each growing season.

Straw management	N rate	Wheat season (kg ha^−1^)				Maize season (kg ha^−1^)			
		N	P_2_O_5_	K_2_O	Straw (wet)	N	P_2_O_5_	K_2_O	Straw (wet)
Straw return (S)	0	0	0	0	7,000	0	0	0	9,000
	360	162	81	81	7,000	198	99	99	9,000
	450	202.5	81	81	7,000	247.5	99	99	9,000
	540	243	81	81	7,000	297	99	99	9,000
	630	283.5	81	81	7,000	346.5	99	99	9,000
	720	324	81	81	7,000	396	99	99	9,000
Straw removal (RS)	0	0	0	0	0	0	0	0	0
	360	162	81	81	0	198	99	99	0
	450	202.5	81	81	0	247.5	99	99	0
	540	243	81	81	0	297	99	99	0
	630	283.5	81	81	0	346.5	99	99	0
	720	324	81	81	0	396	99	99	0

### 2.2 Soil sampling

Before the wheat harvest in 2019, soil samples were randomly collected from each plot at a depth of 0–20 cm. To prevent contamination, the auger was cleaned, wiped with 75% alcohol, and rinsed with sterile water after sampling each plot. The soil cores from each replicate plot were combined into a single composite sample. The soil was sieved through a 2 mm mesh to remove impurities such as roots, fronds, and stones. Each soil sample was then divided into two fractions: one was stored at −80°C for DNA extraction, and the other was air-dried and ground for the determination basic soil properties.

### 2.3 Soil chemical properties analysis

Soil pH was determined using an electromagnetic device (Mettler Toledo Five Easy FE30, Switzerland) at a soil-to-distilled-water ratio of 1:2.5 (weight/volume). SOC was measured using the potassium dichromate oxidation method. Soil NH_4_^+^-N and NO_3_^−^-N were extracted with 2 mol L^−1^ KCl solution (1:5 w/v) and measured using a continuous flow analyzer (San++, SKALAR, Netherlands). Total N (TN) was measured using an automated C/N analyzer (Vario MAX CN, Elementar, Germany). Available phosphorus (AP) was extracted with sodium bicarbonate and determined using the molybdenum-blue method. Available potassium (AK) was extracted with ammonium acetate and measured using a flame photometer (BWB-XP, BWB Technologies, UK).

### 2.4 DNA extraction, PCR amplification, and high-throughput sequencing

DNA was extracted from 0.5 g fresh soil using the E.Z.N.A.R® soil DNA Kit (Omega Bio-tek, Norcross, GA, USA) following the manufacturer’s instructions. DNA concentrations were measured using a NanoDrop™ 2000 spectrophotometer (ThermoScientific, USA). PCR amplification was performed using a Thermocycler PCR system (Gene Amp® 9,700, ABI, USA). Bacterial primers 341F (5’-CCTAYGGGRBGCASCAG-3′) and 806R (5’-GGACTACNNGGGTATCTAAT-3′) were employed to amplify the V3-V4 hypervariable regions of the 16S rRNA gene (Zhu et al., 2022). Primers ITS1F (5’-CTTGGTCATTTAGAGGAAGTAA-3′) and ITS2R (5’-GCTGCGTTCTTCATCGATGC-3′) were used to amplify the fungal rRNA gene in the ITS1 region (Liu J. et al., 2021). PCR was performed in 20 μL reaction volumes containing TransStart Fastpfu DNA Polymerase, forward and reverse primer, double distilled water (ddH_2_O), and DNA template. The PCR cycling conditions were 95°C for 5 min, followed by 27 cycles of 95°C for 30 s, 55°C for 30 s, and 72°C for 30 s. Purified PCR products were extracted from agarose gel using gel electrophoresis, purified with the AxyPrep DNA Gel Extraction Kit (Axygen Biosciences), and quantified using a Quantus™ Fluorometer (Promega) (Yang et al., 2022).

### 2.5 Analysis of the sequencing data

Purified PCR products were pooled in equimolar amounts, and sequenced using an Illumina MiSeq platform by Shanghai BIOZERON Biotechnology Co., Ltd. (Shanghai, China). To accurately delineate different species, OTUs were commonly clustered with a 98.7% similarity threshold using UPARSE (version 7.1^1^) (Ley et al., 2024). The RDP Classifier method assessed bacterial sequences against the SILVA database and fungal sequences against the United States database with a confidence level of 70% (Zheng et al., 2020).

### 2.6 Statistical analysis

Data analyses were performed using R software.^2^ One-way ANOVA assessed the significance of N application rates (*p* < 0.05). Paired t-tests evaluated the significance of straw management treatments (*p* < 0.05). Alpha diversity indices including Shannon index, abundance-based coverage estimate (ACE), and Chao index, were calculated by using QIIME software version 1.8.0. Beta diversity among various N application rates and straw management treatments were evaluated by using Nonmetric multidimensional scaling (NMDS) calculated as Bray–Curtis dissimilarity with “metaMDS” in the vegan package (R version 4.3.2) (Osburn et al., 2019). Mantel tests were conducted to assess the correlations between bacterial and fungal community compositions and diversity with soil properties (e.g., pH, SOC, TN, NO₃^−^-N, NH₄^+^-N, AP, and AK). The analysis was performed in R software using the ‘vegan’ package, following the approach outlined by Ma et al. (2024). The relationships were visualized with the ‘ggcor’ package. Partial least squares path modeling (PLS-PM) was conducted to explore the potential direct and indirect effects of soil properties and microbial communities on crop yield under straw management and N fertilization (Dai et al., 2024). The PLS-PM models were constructed using the ‘plspm’ package in R. The goodness-of-fit index was used to assess the overall predictive performance of the model. A *p*-value of <0.05 was considered to be statistically significant.

## 3 Results

### 3.1 Crop yields and soil chemical properties

**Figure 1:**
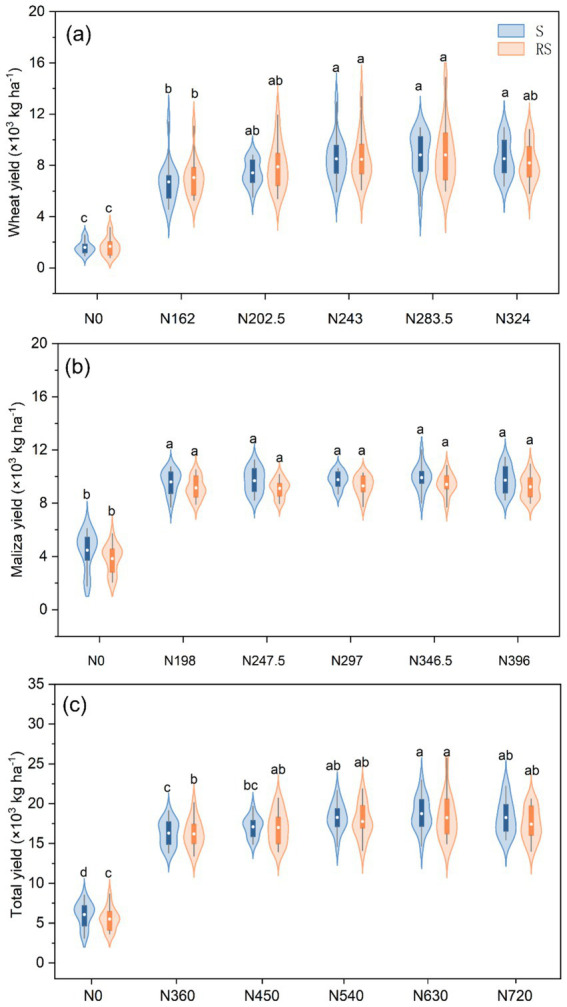
Wheat **(a)**, maize **(b)** and total mean annual yields **(c)** (2014–2018) under different N fertilization levels and straw management. Different lowercase letters indicate significant differences (*p* < 0.05) among different nitrogen levels at the same straw management. “*” denotes significant differences at 0.05 levels among straw return and straw removal. S and RS are straw return and straw removal, respectively.

Annual wheat and maize yields from 2014 to 2018, as influenced by N application rates and straw management, are illustrated in Figure 1. N fertilization significantly increased both wheat and maize yields across all straw management practices compared to the N0 treatment (*p* < 0.05). Straw return increased maize yields by 5–17% relative to straw removal but had no significant effect on wheat yields. When N application rates ranged from 0 to 202.5 kg ha^−1^, straw return decreased wheat yields by 1.78–6.64% compared to straw removal.

The effects of straw management and N application on soil chemical properties are presented in Table 2. Under straw removal, soil pH decreased by 0.51–1.67 units over 12 years as N rates increased from 0 to 720 kg ha^−1^ (*p* < 0.05), and straw return did not mitigate this acidification. SOC content was 25.6–36% higher under straw return than straw removal at equivalent N levels (*p* < 0.05). N fertilization significantly increased TN, NH₄^+^-N, and NO₃^−^-N concentrations (*p* < 0.05) but had no effect on AP or AK levels (*p* > 0.05). Straw return further increased TN and NO₃^−^-N, while decreasing NH₄^+^-N levels (*p* > 0.05). It also increased AK levels (*p* < 0.05), but had no significant effect on AP (*p* < 0.05).

**Table 2:** Changes in soil chemical properties under straw management and nitrogen fertilization.

Straw management	N rate	pH	SOC	TN	NO_3_^−^-N	NH_4_^+^-N	AP	AK
			g kg^−1^		mg kg^−1^			
Straw return (S)	0	7.13Aa	12.02Ab	1.08Ab	3.64Ac	4.22Ab	26.18Aab	205.00Aa
	360	6.44Ab	13.97Aa	1.36Aa	4.00Ab	4.14Ab	33.46Aa	214.17Aa
	450	6.28Ab	13.98Aa	1.29Aab	5.57Ab	4.10Bb	29.03Aab	200.00Aa
	540	5.87Abc	14.38Aa	1.35Aa	7.35Aa	4.66Bb	24.94Ab	188.33Aa
	630	5.62Ac	14.37Aa	1.41Aa	7.17Aa	4.53Bb	22.13Ab	187.50Aa
	720	5.35Ac	15.14Aa	1.35Aa	5.39Ab	6.58Aa	23.13Ab	194.17Aa
Straw removal (RS)	0	7.12Aa	9.25Bb	0.87 Bd	2.26Bb	4.53Ac	18.61Aa	139.17Aa
	360	6.59Ab	10.27Bab	0.93Bcd	3.25Ba	4.78Abc	23.09Aa	134.17Aa
	450	6.33Ab	10.32Bab	0.97Bbcd	3.40Ba	5.97Aabc	23.98Aa	140.83Aa
	540	5.53Ac	11.24Ba	0.99Bbc	3.26Ba	6.31Aab	25.48Aa	149.17Aa
	630	5.56Ac	11.44Ba	1.17Ba	4.02Ba	6.45Aab	20.72Aa	134.17Aa
	720	5.43Ac	11.33Ba	1.08Bab	3.57Ba	7.69Aa	17.38Aa	131.67Aa

Different capital and lowercase letters indicate significant differences between straw management and nitrogen fertilization levels, respectively (*p* < 0.05). SOC, soil organic carbon; TN, total nitrogen; AP, available phosphorus; AK available potassium.

### 3.2 Alpha diversity of soil bacterial and fungal community

**Figure 2:**
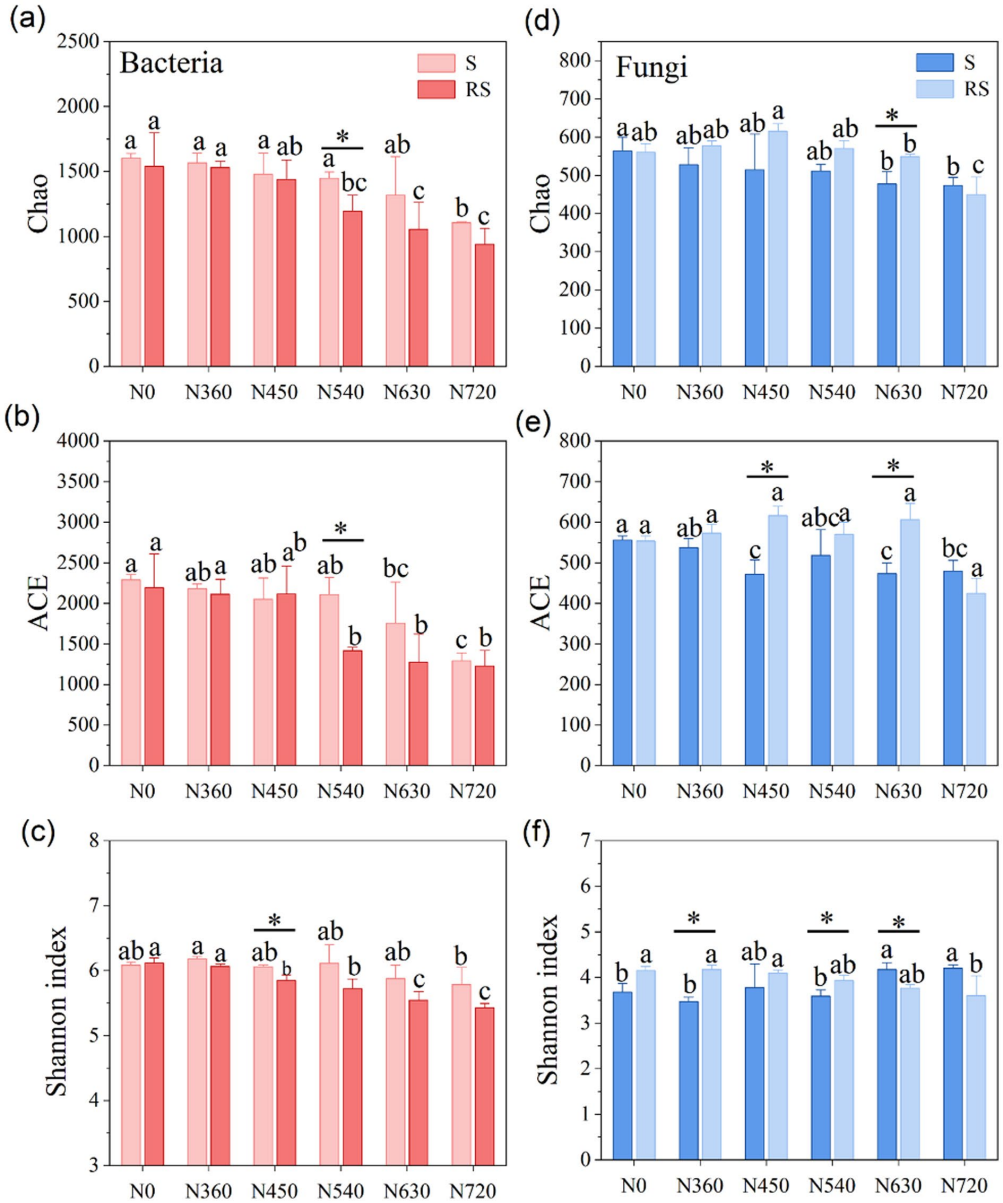
Alpha diversity of soil bacterial (red bar, **a**–**c**) and fungal (blue bar, **d**–**f**) groups under straw management and nitrogen fertilization. “*” denotes significant differences at 0.05 levels among straw return and straw removal. Different lowercase letters indicate significant differences (*p* < 0.05) among different nitrogen levels at the same straw management. S and RS are straw return and straw removal, respectively.

Bacterial richness (Chao and ACE indices) and diversity (Shannon Index) were consistently higher under straw return compared to straw removal, particularly at N540 (Figures 2A–C). However, both straw treatments exhibited reduced bacterial diversity at high N levels (N630, N720), suggesting a threshold beyond which N adversely affects bacterial communities. Fungal richness (Chao and ACE indices) was generally higher under straw removal at most N levels, with significant differences at N630 (Figures 2D,E). Fungal diversity (Shannon index) was greater under straw removal, particularly at N360 and N540 (Figure 2F).

### 3.3 Composition of soil bacterial and fungal community

**Figure 3:**
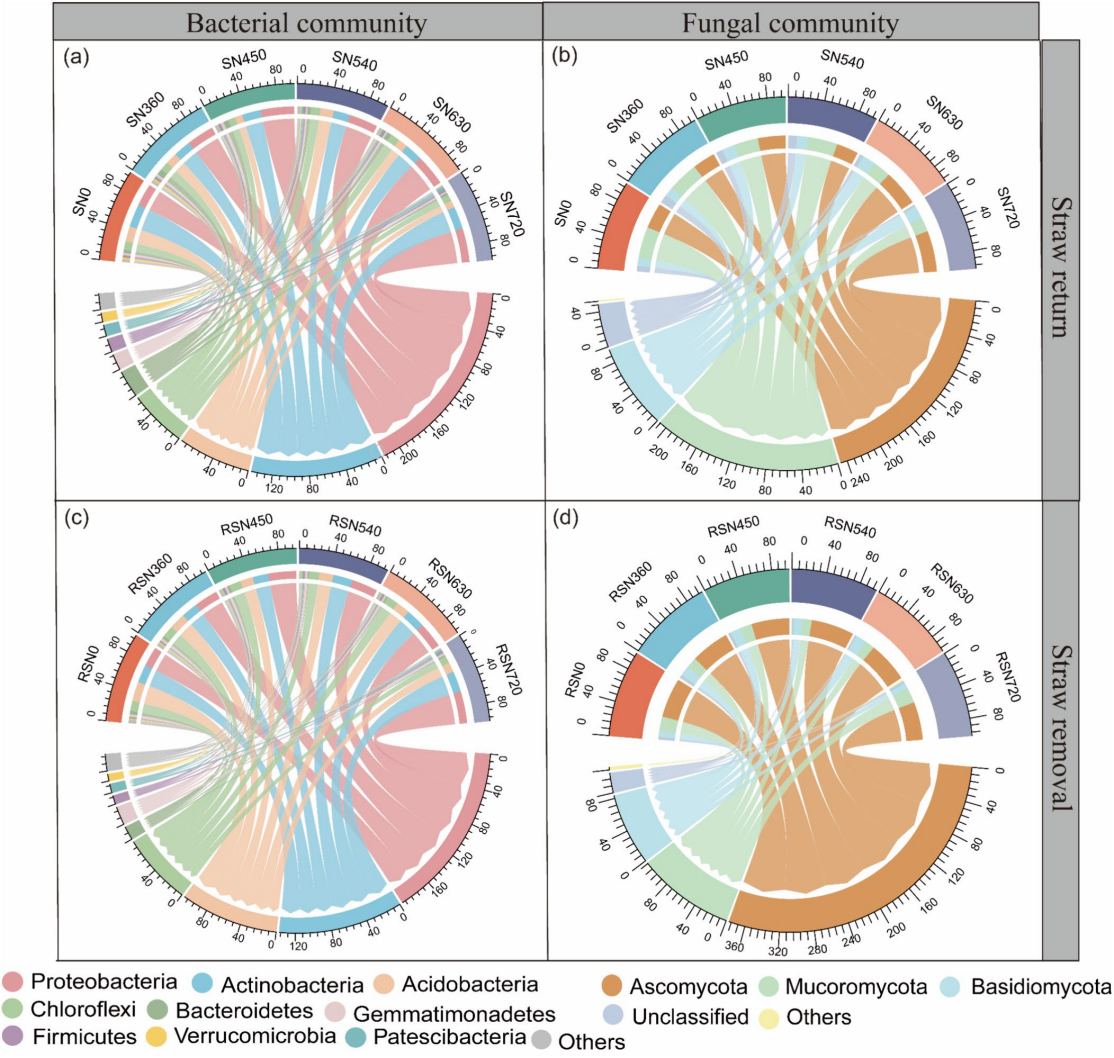
Dominant phylum level of bacterial **(a,c)** and fungal communities **(b,d)** under straw management and nitrogen fertilization.

**Figure 4:**
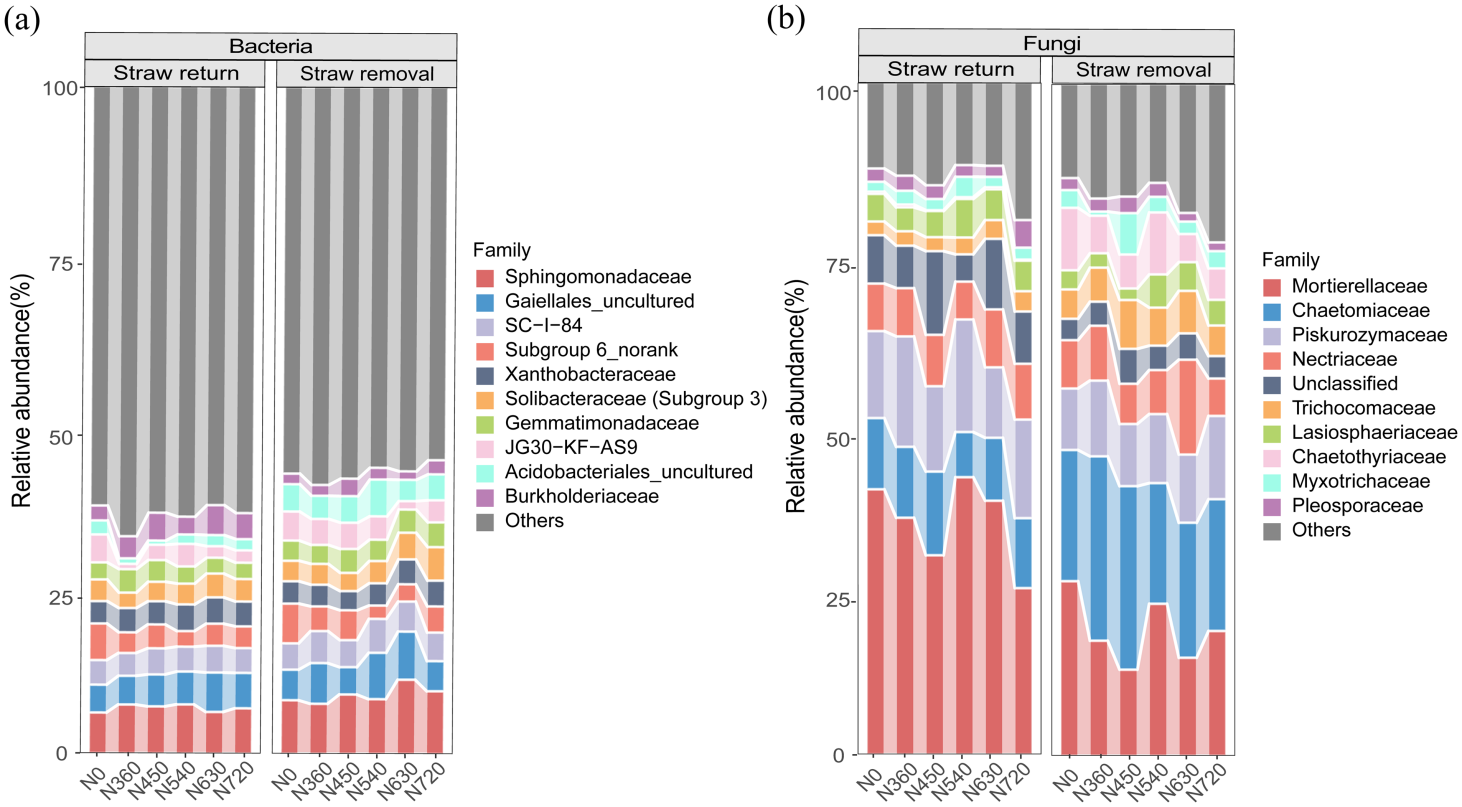
Dominant family level of bacterial **(a)** and fungal communities **(b)** under straw management and nitrogen fertilization.

**Figure 5:**
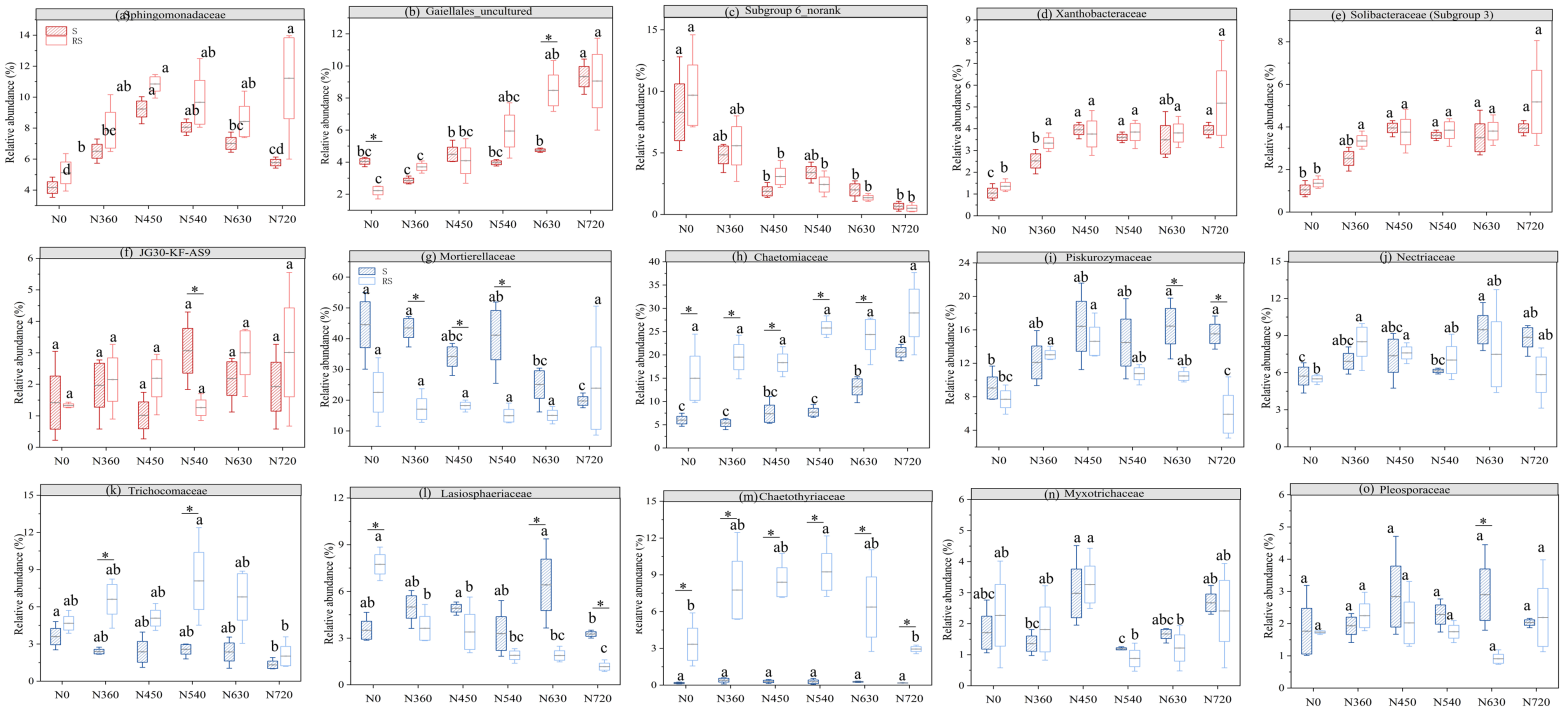
The relative abundances (%) of major bacterial (red box, **a–f**) and fungal (blue box, **g–o**) groups at the family level under straw management and nitrogen fertilization. “*” denotes significant differences at 0.05 levels among straw return and straw removal. Different lowercase letters indicate significant differences (*p* < 0.05) among different nitrogen levels at the same straw management. S and RS are straw return and straw removal, respectively.

Across all treatments, bacterial phyla (Figures 3A,C) and families (Figure 4A) were identified. The dominant phyla were Proteobacteria (24.3–46.9%), Actinobacteria (16.0–33.7%), Acidobacteria (6.7–31.4%), Chloroflexi (5.6–19.5%), and Bacteroidetes (1.1–8.5%). In Proteobacteria, the predominant families included Sphingomonadaceae (4.2–11.2%), and Xanthobacteraceae (0.5–9.7%). N fertilization significantly increased the abundance of Xanthobacteraceae compared to the N0 treatment (*p* < 0.05, Figure 5D). Sphingomonadaceae initially increased at moderate N levels (N450), but declined at higher rates (N540, N630) under straw return (*p* < 0.05, Figure 5A). In Actinobacteria, Gaiellales_uncultured (2.2–9.3%) was a dominant family, showing a significant increase with higher N rates (*p* < 0.05, Figure 5B). In Acidobacteria, the predominant families included Subgroup_6_norank (0.6–8.3%), Solibacteraceae (Subgroup 3) (1.0–4.6%), and JG30-KF-AS9 (1.0–4.8%). Subgroup_6_norank decreased with increasing N rates (Figure 5C), whereas Solibacteraceae (Subgroup 3) displayed a gradual increase (Figure 5E). In contrast, JG30-KF-AS9 showed no consistent trends across treatments (Figures 5F).

Among the fungal phyla, Ascomycota (25.3–77.1%), Mucoromycota (8.70–55.0%), and Basidiomycota (9.90–19.3%) were dominant (Figures 3B,D). The fungal families were also identified (Figure 4B). In Ascomycota, the predominant families included Chaetomiaceae (5.29–29.0%), Nectriaceae (5.56–9.55%), Trichocomaceae (1.40–8.15%), Lasiosphaeriaceae (1.11–7.75%), Chaetothyriaceae (0.17–9.20%), Pleoaporaceae (0.92–2.88%), and Myxotrichaceae (1.14–3.24%). Under straw return, the abundance of Chaetomiaceae showed a gradual increase with rising N application rates (*p* < 0.05) (Figure 5H). In contrast, Trichocomaceae and Lasiosphaeriaceae decreased with higher N rates (*p* < 0.05) (Figures 5K,L). Chaetothyriaceae was more abundant under straw removal across all N levels but showed no consistent response to N fertilization (Figure 5M). In Mucoromycota, Mortierellaceae (10.6–43.5%) was dominant, showing a decreasing trend with nitrogen application under straw return (Figure 5G). In Basidiomycota, Piskurozymaceae (0.86–2.88%) was the predominant family. It displayed a sharp reduction at high N levels under straw removal, suggesting sensitivity to excessive N fertilization, whereas it gradually increased under straw return (Figure 5O).

**Figure 6:**
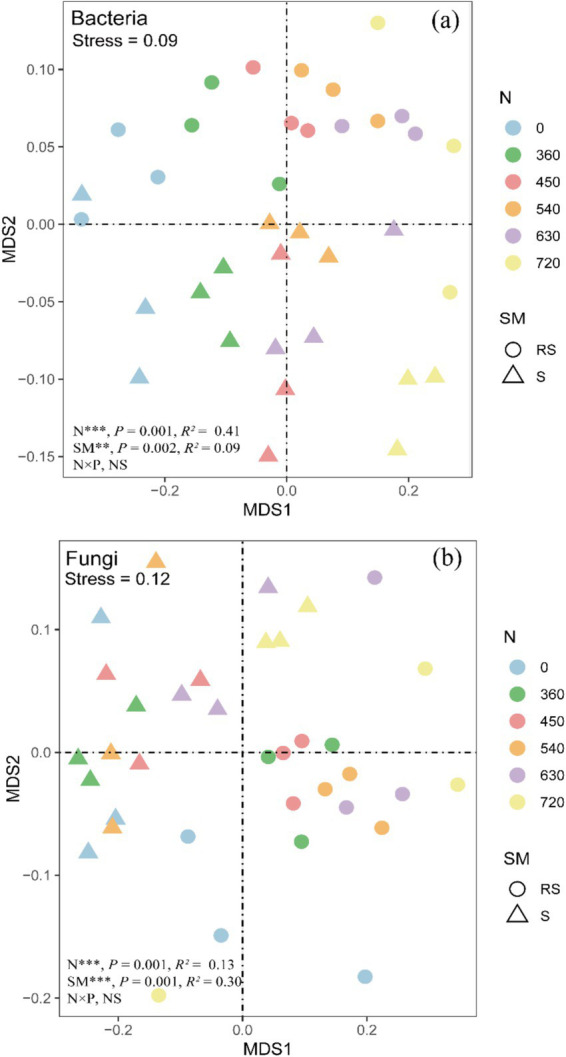
NMDS of bacterial **(a)** and fungal communities **(b)** at the family level under straw management and nitrogen fertilization. *Significant at *p* < 0.05; **Significant at *p* < 0.01; ***Significant at *p* < 0.001; NS, not significant. N, SM, S and RS are nitrogen levels, straw management, straw return and straw removal, respectively.

To evaluate the impact of N fertilization and straw management on soil microbial community composition, performed NMDS analysis based on Bray-Curtis dissimilarities at the family level for bacterial and fungal communities. The results revealed that N fertilization significantly influenced bacterial (R^2^ = 0.41, *p* = 0.001) and fungal community composition (R^2^ = 0.13, *p* = 0.001) (Figure 6). Similarly, straw management led to notable changes in bacterial (R^2^ = 0.09, *p* = 0.002) and fungal communities (R^2^ = 0.30, *p* = 0.001). However, the interaction between N fertilization and straw management did not show a significant effect on microbial community composition.

### 3.4 Relationships between microbial communities, soil properties, and crop yields

**Figure 7:**
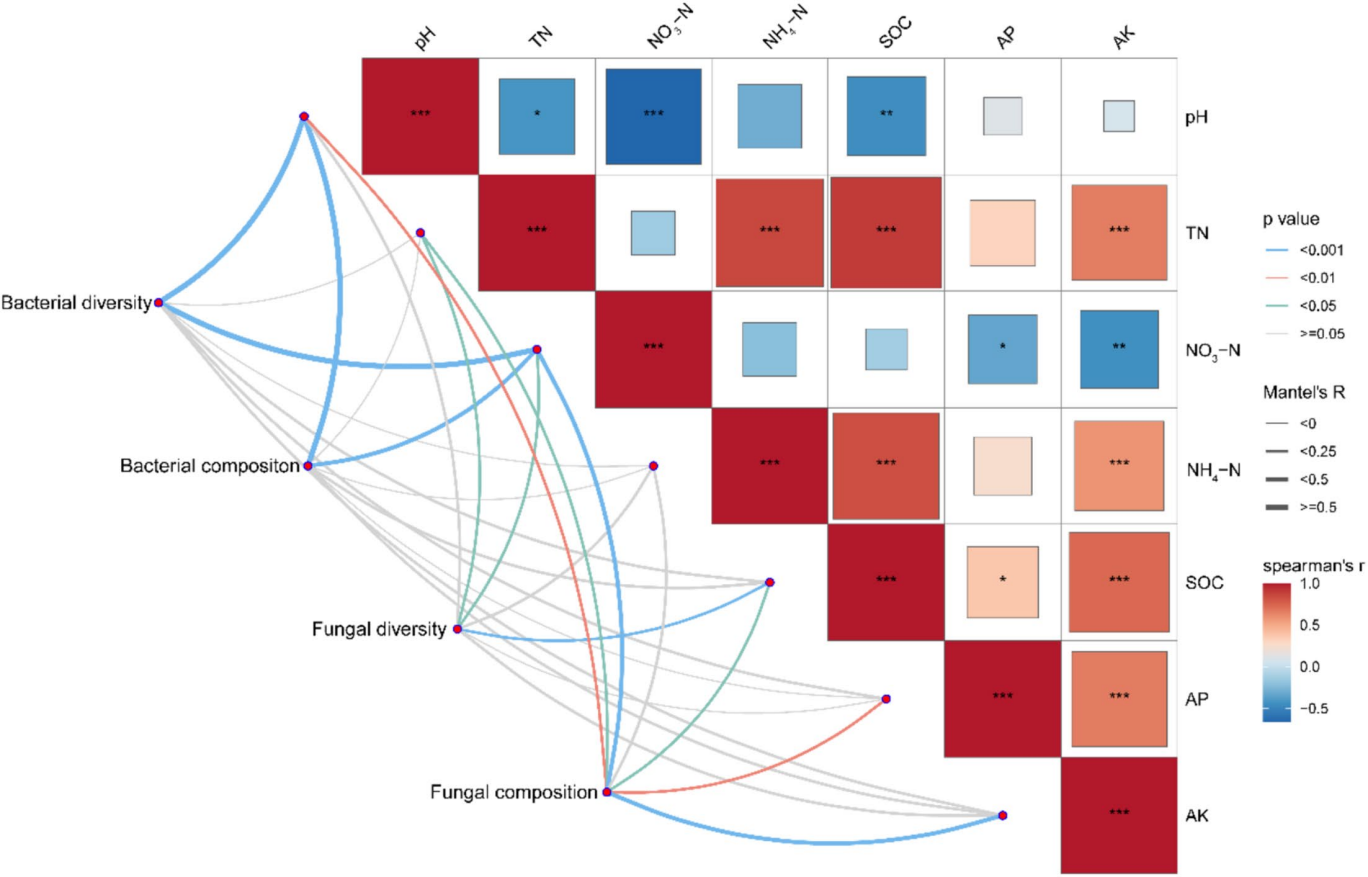
A Mantel test between the bacterial community, fungal community and soil properties. Mantel’s R and *p* values are indicated based on the color and the width of the connecting lines as specified in the figure legend. TN, total nitrogen; SOC, soil organic carbon; AP, available phosphorus; AK available potassium. *Significant at *p* < 0.05; **Significant at *p* < 0.01; ***Significant at *p* < 0.001.

The Mantel tests revealed that soil bacterial and fungal community structures were significantly correlated with multiple soil properties (Figure 7). Bacterial diversity was positively correlated with soil pH (*p* < 0.001), but negatively associated with NH₄^+^-N (*p* < 0.001). Fungal diversity showed weak correlations with most soil properties but was significantly influenced by SOC and TN (*p* < 0.01). Bacterial composition showed strong correlations with soil pH and NO₃^−^-N (*p* < 0.001), whereas fungal composition was primarily affected by soil pH, SOC, NO₃^−^-N, TN, AP, and AK (*p* < 0.05).

**Figure 8:**
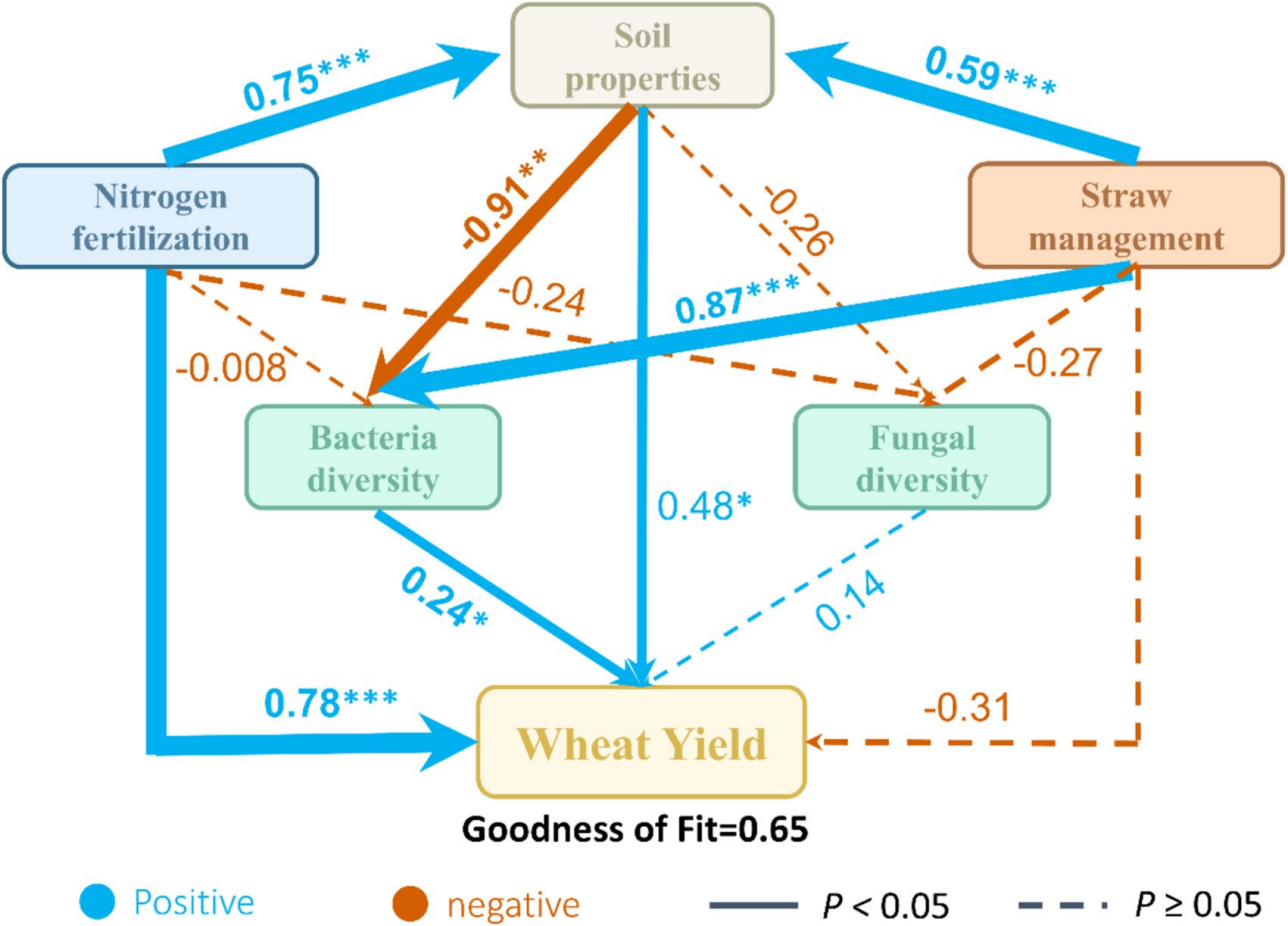
Partial least squares path modeling (PLS-PM) analysis of the relationships between nitrogen fertilization, straw management, soil properties, bacteria and fungi diversity, and wheat yield. Soil properties include pH, soil organic carbon, total nitrogen, NO_3_^−^-N, NH_4_^+^-N, available phosphorus and available potassium. Bacteria diversity: Chao, ACE and Shannon index. Fungal diversity: Chao, ACE and Shannon index. Observed or latent variables are illustrated in box. Positive and negative effects are represented by blue and orange arrows, respectively. Path coefficients that were not significantly (*p* > 0.05) are shown as dashed lines; *p < 0.05, **p < 0.01, and ***p < 0.001. The goodness-of-fit was used to assess the model.

To further explore the direct and indirect effects on the soil properties and bacterial and fungal communities on wheat and maize yields under straw management and nitrogen fertilization. The PLS-PM analysis (goodness of fit = 0.65; Figure 8) indicated that N fertilization had a strong direct positive effect on wheat yield (path coefficient = 0.78, *p* < 0.001) and soil properties (0.75, *p* < 0.001). Straw management positively influenced soil properties (0.59, *p* < 0.001) and bacterial diversity (0.87, *p* < 0.001), indirectly enhancing wheat yield through improved soil properties and bacterial diversity. Similarly, N fertilization indirectly increased wheat yield by enhancing both soil properties (0.91, *p* < 0.01) and bacterial diversity (0.24, *p* < 0.05).

## 4 Discussion

### 4.1 Effects of straw management and N fertilization on crop yields and soil properties

This study demonstrated that N fertilization significantly increased wheat and maize yields, but excessive application reduced yield benefits beyond 283.5 kg N ha^−1^ for wheat and 346.5 kg N ha^−1^ for maize (Figure 1). This yield decline at high N levels is likely due to reduced N use efficiency, as excessive N fertilization can induce nutrient imbalances, inhibit root growth, and increase N loss through leaching or volatilization (Dong and Lin, 2020; Huang et al., 2023; Ren et al., 2024). Consistent with previous studies, maize yield benefited more from straw return compared to wheat (Islam et al., 2022), possibly due to differences in root architecture and seasonal temperature variations that accelerate wheat straw decomposition. In contrast, wheat yield declined under straw return at low to moderate N levels, likely due to delayed N mineralization and physical constraints from undecomposed maize straw (Kong, 2014; Qi et al., 2015).

Soil properties were significantly altered by both N fertilization and straw management. High N rates caused significant soil acidification (pH decline of 0.51–1.67 units; Table 2), which is attributed to ammonium nitrification and H^+^ release (Tian and Niu, 2015). Despite the expected buffering effect of organic inputs, straw return did not mitigate this acidification. However, straw return substantially increased SOC and TN contents, which enhances soil fertility and structural stability (Li et al., 2018). These improvements in soil organic matter contribute to better soil aeration and aggregate stability, potentially improving long-term sustainability in Vertisol cropping systems (Zhao et al., 2020).

### 4.2 Effects of straw management and N fertilization on soil microbial diversity and composition

Long-term N fertilization and straw management significantly influenced soil microbial diversity and composition. Straw return generally enhanced bacterial richness and diversity, especially at moderate N levels such as N540. This suggests that straw incorporation provides a more stable carbon source, fostering bacterial proliferation and functional diversity (Tian et al., 2017; Shen et al., 2023; Zhang et al., 2024). The positive effects of straw return aligns with previous studies, which have shown that the addition of organic matter enhances microbial diversity by providing labile carbon substrates, and stimulating bacterial metabolism (Xu et al., 2023). However, at higher N levels (N630, N720), bacterial diversity declined under both straw return and removal, suggesting that high N inputs may exceed microbial nutrient thresholds, leading to nutrient imbalances and competitive exclusion (Figures 2A–C). High N inputs may favor fast-growing copiotrophic taxa such as Proteobacteria, which rapidly assimilate nutrients, outcompeting oligotrophic groups such as Acidobacteria, known for their preference for nutrient-poor conditions (Chen et al., 2023; Li et al., 2019). This shift in microbial community structure under high N fertilization reflects a loss of microbial diversity and potential disruptions in microbial-driven soil functions.

Unlike bacteria, fungal diversity exhibited an opposite trend, being generally higher under straw removal across most N levels, with significant differences at N630 (Figures 2D–F). This finding contrasts with the findings of Yang et al. (2022) who reported increased fungal diversity with straw return. One possible reason could be the availability of substrates and competition dynamics between bacterial and fungal taxa. As shown in Figure 5, Chaetomiaceae-a key decomposer of plant residues-increased with rising N levels under straw return, while Trichocomaceae and Lasiosphaeriaceae declined at higher N rates (*p* < 0.05). This suggests that fungal taxa might exhibit distinct responses depending on N availability and the composition of decomposing organic matter. Additionally, bacterial diversity was significantly higher under straw return, particularly at N540 (Figures 2A–C), indicating that bacteria might have outcompeted fungi in utilizing straw-derived carbon substrates. The negative correlation between bacterial diversity and NO₃^−^-N (*p* < 0.001, Figure 7) further supports the notion that shifts in soil chemistry could influence microbial interactions.

The NMDS analysis further confirmed the significant effects of N fertilization and straw management on bacterial and fungal communities (Figure 6). Among bacterial phyla, Proteobacteria, Actinobacteriota, Acidobacteria, and Ascomycota were dominant across all treatments, with varying responses to straw management and N fertilization. Proteobacteria, particularly Sphingomonadaceae and Xanthobacteraceae, exhibited a significant increase with increasing N rates (Figures 4A, 5A,D), consistent with their copiotrophic nature and ability to utilize labile carbon compounds released from decomposing straw (Zhang et al., 2021; Dang et al., 2022). However, at high N levels (N630, N720), Sphingomonadaceae abundance declined under straw return (Figure 5A), suggesting excessive N fertilization effects or shifts in substrate availability limiting their growth. Conversely, Acidobacteria, particularly Subgroup_6_norank and JG30-KF-AS9, exhibited a decline with increasing N levels (Figures 5C,F). Acidobacteria are oligotrophic taxa, often thriving in low-nutrient conditions and being negatively affected by high N inputs (Kalam et al., 2020; Gu et al., 2023). However, Solibacteraceae (Subgroup 3), another Acidobacteria subgroup, showed an increasing trend with rising N levels (Figure 5E), possibly indicating niche differentiation within Acidobacteria, where some taxa can adapt to increased nutrient availability while others are outcompeted.

Fungal communities responded differently to N fertilization and straw return compared to bacteria. Ascomycota, Mucoromycota, and Basidiomycota were the dominant fungal phyla (Figures 3B,D). Within Ascomycota, key families exhibited distinct trends: Chaetomiaceae increased with rising N application rates under straw return (*p* < 0.05, Figure 5H), while Trichocomaceae and Lasiosphaeriaceae showed significant declines at higher N levels (*p* < 0.05, Figures 5K,L). These responses suggest that certain fungal taxa, particularly Chaetomiaceae, may be well adapted to high N environments, whereas others are more sensitive to excessive N inputs. Interestingly, Chaetothyriaceae was consistently more abundant under straw removal but showed no clear response to N levels (Figure 5M), indicating that factors other than N availability, such as soil aeration or substrate recalcitrance, may influence its distribution. Mucoromycota, represented predominantly by Mortierellaceae, exhibited a declining trend with increasing N application under straw return (Figure 5G). This suggests that Mucoromycota, known for their role in organic matter decomposition, may be negatively affected by high N levels, possibly due to shifts in substrate composition or competition with bacteria (Dong et al., 2023; Song B. et al., 2023; Song T. et al., 2023). Basidiomycota, particularly Piskurozymaceae, displayed a sharp reduction under high N fertilization with straw removal, while its abundance increased under straw return (Figure 5O), highlighting potential interactions between N inputs and organic matter availability in shaping fungal community structure.

### 4.3 Linkages between soil microbial communities, soil properties, and crop yield

The findings of this study demonstrate that N fertilization significantly increased wheat yield and soil properties (*p* < 0.001, Figure 8), reinforcing its essential role in crop production. However, high N fertilization resulted in soil acidification (Table 2), which negatively affected bacterial diversity (Figure 7). This pattern aligns with previous studies suggesting that soil acidification can suppress pH-sensitive bacterial taxa, potentially disrupting key microbial processes such as organic matter decomposition and nutrient cycling (Tian and Niu, 2015; Gayan et al., 2023; Hu et al., 2024). Straw return significantly enhanced SOC and TN (Table 2), which positively influenced bacterial diversity (Figure 8). The microbial-mediated benefits of straw return were particularly evident at moderate N levels (N450–N540), where bacterial diversity and crop yields were optimized (Figures 1, 2A–C). The relative increase in Xanthobacteraceae and Gaiellales_uncultured (Figures 5B,D) under these conditions suggests an improved microbial capacity for organic matter turnover and nitrogen cycling, which likely contributed to enhanced soil fertility (Zhao et al., 2020; Liu N. et al., 2021). Interestingly, fungal diversity exhibited weaker correlations with crop yield (Figure 8), indicating that bacterial-driven transformations in soil properties had a more direct influence on productivity. The Mantel test further revealed that fungal diversity was significantly associated with SOC and TN (Figure 7), suggesting that fungal contributions were more linked to long-term soil organic matter stabilization rather than immediate nutrient availability (Pérez-Izquierdo et al., 2021; Liu et al., 2024). For example, Chaetomiaceae increased with rising N levels under straw return (Figure 5H), while Trichocomaceae and Lasiosphaeriaceae decreased (Figures 5K–L), indicating shifts in fungal decomposer communities that could alter carbon cycling dynamics.

The PLS-PM analysis revealed that bacterial diversity indirectly enhanced wheat yield via improved soil properties (Figure 8), underscoring the importance of bacterial-mediated soil fertility in this system. In contrast, fungal community shifts did not exhibit a direct correlation with crop yield, suggesting that bacterial contributions to N transformations and organic matter decomposition played a more dominant role in driving productivity. These findings highlight the need for balanced N fertilization strategies that optimize microbial functions while mitigating soil degradation. While moderate N application (N450-N540) with straw return supported microbial diversity and improved soil conditions, excessive N input (N630-N720) negatively affected bacterial diversity and altered fungal community composition. Future research should focus on functional microbial traits and their roles in nutrient transformations, providing deeper insights into microbial-driven pathways for improving soil health and crop resilience.

## 5 Conclusion

This study evaluates the combined effects of straw management and N fertilization on crop yields, soil properties, and microbial communities in a wheat-maize cropping system on Vertisol. Microbial community composition responded differently to N fertilization and straw management. Moderate N levels (N450-N540) with straw return enhanced bacterial diversity, particularly Sphingomonadaceae and Solibacteraceae (Subgroup 3), while high N levels (N630, N720) reduced bacterial diversity. In contrast, fungal diversity was generally higher under straw removal, with Chaetomiaceae increasing under straw return, whereas Mortierellaceae and Trichocomaceae declined at higher N rates. Mantel tests revealed that SOC and N availability (NO₃^−^-N, TN) are key drivers shaping microbial communities, while soil pH plays a crucial role in bacterial diversity and composition. PLS-PM analysis further confirmed that N fertilization directly increased wheat yield, whereas straw return improved soil fertility and microbial diversity, indirectly supporting yield sustainability.

## Data Availability

The datasets presented in this study can be found in online repositories. The names of the repository/repositories and accession number(s) can be found in the article/supplementary material.

## Footnotes

^1^ http://drive5.com/uparse/

^2^ https://www.r-project.org/
